# Delivery of magnetic micro/nanoparticles and magnetic-based drug/cargo into arterial flow for targeted therapy

**DOI:** 10.1080/10717544.2018.1497106

**Published:** 2018-12-06

**Authors:** Mohammad K. D. Manshadi, Mahsa Saadat, Mehdi Mohammadi, Milad Shamsi, Morteza Dejam, Reza Kamali, Amir Sanati-Nezhad

**Affiliations:** a Department of Mechanical and Manufacturing Engineering, University of Calgary, Calgary, Alberta, Canada;; b Department of Chemical Engineering, College of Engineering, Shahid Bahonar University of Kerman, Kerman, Iran;; c Department of Biological Science, University of Calgary, Calgary, Alberta, Canada;; d Center for Bioengineering Research and Education, University of Calgary, Calgary, Alberta, Canada;; e Department of Petroleum Engineering College of Engineering and Applied Science, University of Wyoming, Laramie, WY, USA;; f Department of Mechanical Engineering, Shiraz University, Shiraz, Iran

**Keywords:** Magnetic drug targeting, drug/cargo delivery, artery, cancer therapy, computational fluid dynamics, numerical simulation

## Abstract

Magnetic drug targeting (MDT) and magnetic-based drug/cargo delivery are emerging treatment methods which attracting the attention of many researchers for curing different cancers and artery diseases such as atherosclerosis. Herein, computational studies are accomplished by utilizing magnetic approaches for cancer and artery atherosclerosis drug delivery, including nanomagnetic drug delivery and magnetic-based drug/cargo delivery. For the first time, the four-layer structural model of the artery tissue and its porosity parameters are modeled in this study which enables the interaction of particles with the tissue walls in blood flow. The effects of parameters, including magnetic field strength (MFS), magnet size, particle size, the initial position of particles, and the relative magnetic permeability of particles, on the efficacy of MDT through the artery walls are characterized. The magnetic particle penetration into artery layers and fibrous cap (the covering layer over the inflamed part of the artery) is further simulated. The MDT in healthy and diseased arteries demonstrates that some of the particles stuck in these tissues due to the collision of particles or blood flow deviation in the vicinity of the inflamed part of the artery. Therefore the geometry of artery and porosity of its layers should be considered to show the real interaction of particles with the artery walls. Also, the results show that increasing the particles/drug/cargo size and MFS leads to more particles/drug/cargo retention within the tissue. The present work provides insights into the decisive factors in arterial MDT with an obvious impact on locoregional cancer treatment, tissue engineering, and regenerative medicine.

## 1. Introduction

Magnetic drug targeting (MDT) is one of the active delivery methods which attracted the attention of researchers for applications in gene therapy, controlling cellular function, magnetic imaging, tissue engineering, and regenerative medicine (Al-Jamal et al., 2016; Alexiou et al., 2011; Pankhurst et al., 2009; Shamsi et al., 2018a,b; Tietze et al., 2015). Drugs loaded on magnetizable particles (e.g. iron oxide) in a wide range of diameters have been synthesized using different methods. Ferroferric oxide magnetic microparticles have been prepared widely by coprecipitation methods (Xiao et al., 2016). Small nanoparticles (<10 nm) have been fabricated using the precipitation of iron salts in aqueous media. Medium sized nanoparticles (10–30 nm) have been obtained mostly from high-temperature decomposition of organic precursors (Indira & Lakshmi, 2010; Roca et al., 2009). Larger nanoparticles with the diameter value in the range between 25 to 300 nm have been produced by oxidation of an iron (II) salt using mild oxidant methods (Roca et al., 2009). Biocompatible magnetic drugs are concentrated at a specific tissue site by bonding drugs to magnetic particles, injecting them into blood stream or targeted tissue, and steering them to the desired location by external magnetic fields (Cherry & Eaton, 2014; Modarres et al., 2018; Xu et al., 2005). The magnetic field could be generated by an external magnet or an implanted magnetic source (typically a stent, wire, or spherical seed) to target the desired place deep within the body (Cregg et al., 2012; Rukshin et al., 2017).

In some MDTs, magnetic field pulls the drug particles from blood vessels into the vessel tissues (Nacev et al., 2011) with the potential application in vascular normalization therapy to improve perfusion and drug delivery to hypoperfused tumors (Gkretsi et al., 2017). Among blood vessels, MDT through arteries can be particularly used for cancer therapies. The hepatic artery for the treatment of liver tumor (Jeon et al., 2016), the femoral artery for treating hind limb tumor (Alexiou et al., 2011), and the carotid artery for brain tumor treatment (Chertok et al., 2010) have been the selected arteries. The primary advantage of releasing drugs into the artery closest to the desired delivery site is keeping the carriers away from the liver before reaching the target (Alexiou et al., 2006). The performance of the particle clusters for MDT through arteries has also been investigated to elucidate the role of different parameters such as velocity profile, bulk flow velocity, particle size, magnetic field, releasing points, Brownian motion, particles diffusion, non-Newtonian blood models on steering particles to the desired site (Asfer et al., 2015; Cherry & Eaton, 2014; Cherry et al., 2010; Gitter & Odenbach, 2013; Hamdipoor et al., 2018; Lunnoo & Puangmali, 2015; Rukshin et al., 2017; Yue et al., 2012). It was demonstrated that velocity profile has no significant effect on particle retention but the increase in the bulk flow velocity results in a declines of particle trapping (Asfer et al., 2015; Cherry et al., 2010; Yue et al., 2012). Therefore, trapping efficiency is high in a small vessel with low blood velocity (Lunnoo & Puangmali, 2015). Furthermore, increasing the particle diameter and magnetic field strength (MFS) enhances the magnetophoretic force and improves the particle capture efficiency (Asfer et al., 2015; Cherry et al., 2010; Lunnoo & Puangmali, 2015; Yue et al., 2012). Besides, the releasing point of particles also affects their capturing efficacy. Particles released next to the tumor showed a higher trapping efficiency while releasing the particles very close to the tumor resulted in a less time for the magnet to trap the particles and therefore less performance in controlled release to the targeted site (Rukshin et al., 2017). The Brownian motion and diffusion of particles through the blood are also influential parameters on MDT of smaller particles where the particles with diameters smaller than 20 nm are significantly affected by Brownian motion (Yue et al., 2012). The collision of particles with circulating red blood cells also enhances their Brownian motion limiting the magnetic control effectiveness (Cherry & Eaton, 2014; Rukshin et al., 2017). To increase targeting efficiency, new MDT strategies have been developed to control particles direction and increase drug concentration at the desired site (Cherry & Eaton, 2014; Gitter & Odenbach, 2013; Hamdipoor et al., 2018). Drug/cargo delivery is one of the recent advances in this area. For drug/cargo delivery, the drug is loaded on micro/nanomotors and steered to the targeted site (Gao & Wang, 2014). Some of these micro/nanomotors are fuel free, and external sources such as magnetic field are employed to drive them toward the destination (Gao et al., 2012). Magnetic drug/cargo delivery has proven its potential for cancer therapy (Gao et al., 2018). Various methods have been employed for the fabrication of micro and nanomotors include roll-up technology that is based on traditional thin-film deposition (Xu et al., 2017; Zhao et al., 2014), glancing angle deposition (Ghosh & Fischer, 2009), direct laser writing (Tottori et al., 2012), membrane template-assisted electrodeposition (Wang, 2013), and spiral water-conducting (Gao et al., 2014).

Besides cancer therapy, cargo/drug delivery has also been employed for the therapy of cardiovascular diseases (Binsalamah et al., 2012). For example, atherosclerosis cardiovascular disease is an inflammatory disease associated mainly with large and medium-sized vessels from approximately 3 mm external diameter arteries up to the aorta arteries (Davies & Woolf, 1993; Perrotta & Aquila, 2015; Ross & Glomset, 1973). Inflammation of artery intima results from increased concentration of phospholipids and cholesterol, proliferation of smooth muscle cells, and low-density lipoprotein (Anogeianaki et al., 2011; Bose & Banerjee, 2015; Bozsak et al., 2014; Mirza et al., 2017; Priyadharshini & Ponalagusamy, 2018; Soleimani et al., 2018). The smooth muscle cells contribute to fibrous cap formation and protect the core of inflammation (Lee et al., 2017). The primary consequence of artery inflammation is plaque rupture due to low resistance of the plaques to hemodynamic blood pressure and shear stress (Li et al., 2006).

**Figure 1.**
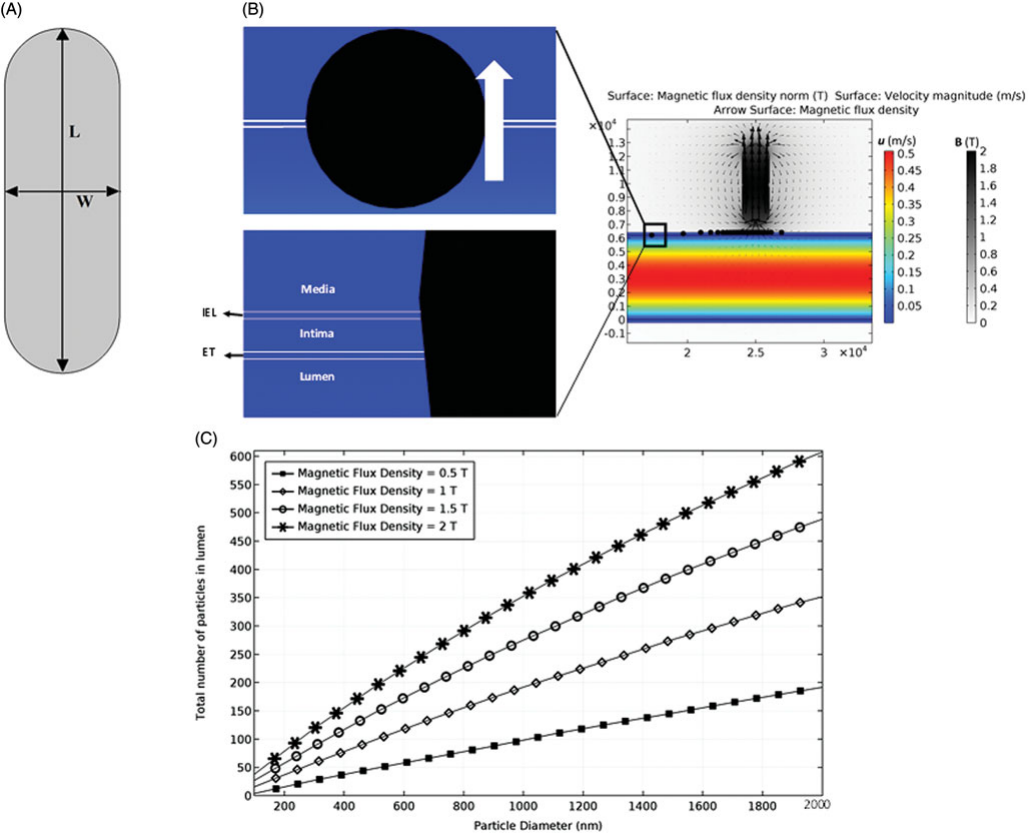
The permanent magnet and its magnetic field. (A) The employed magnet with a length of *L* = 6 mm and a width of *W* = 2 mm. (B) The effect of permanent magnet on particles retention. (C) The effects of magnetic field strength (MFS) and particle diameter on the efficacy of particle trapping.

Given the key role of arteries in drug delivery for several cancers and inflammation diseases, developing new models for characterizing the MDT performance through the artery is of high importance. Among various models of the drug delivery through arteries, the four-layer artery model (where the arterial wall is divided into endothelium (ET), intima, internal elastic lamina (IEL), and media) is the most realistic one (Yang & Vafai, 2006) (Supplemental Figure S1^Figure 1.^; Yang & Vafai, 2006). However, the magnetic drug delivery performance through layers of the luminal artery has not been investigated thus far. Also, MDT-based drug penetration through the fibrous cap of the artery for healing of artery inflammation has not yet been studied.

In this work, we examine MDT through a four-layer model of the artery. The delivery of both clusters of drug particles and single drug/cargo into the luminal artery for curing inflammation, and their translocation through the artery layers are studied. Particle clusters and single micro drug/cargo particles are traced within the luminal artery and their response to external magnetic fields is characterized. MDT for the therapy of luminal artery inflammation while considering the fibrous cap tissue is also explored. Since the maximum limit of magnetic exposure for human body should not exceed 0.4 (T) (International Commission on Non-Ionizing Radiation Protection, 2009), we used a 2 (T) permanent magnet with a deep influence of the applied magnetic field below 0.4 (T). Following the validation of the numerical model, several decisive parameters, including MFS, magnet size, particle size, drug/cargo size, and magnetic relative permeability of particles are studied to find their effects on MDT performance where the artery porosity is considered.

## 2. Methods

### 2.1. Governing equations

Parameters such as blood flow, magnetic field, and properties of micro/nanoparticles play crucial roles in MDT performance. Multiple physics of fluid flow, Gauss law, and particle tracing are combined to investigate the efficacy of MDT. Conservation equations of mass and momentum (Navier–Stokes) are solved (Equations (1) and (2)) to determine blood flow through the vessel (Kamali et al., 2016).

(1) $$\rho \nabla .\left(u\right)=0,$$

(2) $$\rho \left(u\cdot \nabla \right)u=-\nabla P+\mu \nabla \cdot \left(\nabla u\right)+F,$$

where *ρ* (kg/m^3^) is the blood density, ***u*** (m/s) is the blood velocity vector, *µ* (Pa.s) is the blood dynamic viscosity, *P* (Pa) is pressure, and ***F*** (N/m^3^) is the external volume force. For the blood flow through tissues, the tissue porosity and blood permeability are applied in Darcy-Brickman momentum Equation (3) (Andreas et al., 2008).

(3) $$\frac{1}{\phi }\rho \left(u\cdot \nabla \right)\frac{u}{\phi }=-\nabla P+\frac{\mu }{\phi }\nabla \cdot \left(\nabla u\right)-\frac{\mu }{k}u+F,$$

where *φ* represents porosity and *k* (m^2^) denotes tissue permeability. A permanent magnetic field is exerted in the target area to trap particles. The resultant magnetic field is calculated from $\nabla \cdot \left(B\right)=0$ (Lunnoo & Puangmali, 2015) where ***B*** (T) is the applied magnetic field. The magnetic field around the permanent magnet is obtained from $B=\mu_{0}\mu_{r}\left(H+M\right)\;$where *µ*_0_ (H/m) is magnetic permeability of space, *µ_r_* is the relative magnetic permeability, ***H*** (A/m) is the induced magnetic field, and ***M*** is magnetization of the material. For the blood and tissue, magnetization is zero and therefore ***B*** (T) is simplified as $B=\mu_{0}\mu_{r}H$.

Newton’s second law in Equation (4) is used to trace particles in a media.

(4) $$\frac{d(m_{p}V)}{dt}=\sum F_{p},$$

where *m*_p_ (kg) is particle mass, ***V*** (m/s) is particle velocity, and *F*_p_ (N) is the acting force on particles. The rate of momentum change (mv) for particles equals the sum of acting forces, including the drag and magnetophoretic forces. Brownian motion is neglected since particles diameter is selected larger than 50 nm (Lunnoo & Puangmali, 2015). To calculate drag force, a well-known accurate Schiller-Naumann model is used which is defined in Equations (5)–(7) (Loth, 2008).

(5) $$F_{d}=\frac{3\mu C_{D}{\text{Re}}_{r}}{4\rho_{p}d_{p}^{2}}m_{p}(u-V),$$

(6) $$C_{D}=\frac{24}{{Re}_{b}}\left(1+0.15{Re}_{b}^{0.687}\right),$$

(7) $${\text{Re}}_{b}=\frac{\rho \left\|\mathrm{|u}-\right.\left.\mathrm{V|}\right\|d_{p}}{\mu },$$

where *ρ*_p_ (kg/m^3^) is particle density, Re_b_ is blood Reynolds number, and *d*_p_ (m) is particle diameter. The ferroparticles can be magnetized in an external magnetic field and attracted to the magnet. The generated magnetophoretic force is defined as in Equation (8).

(8) $$F_{m}=2\pi r_{p}^{3}\mu_{0}\mu_{r}\left(\frac{\mu_{r,\;p}-\mu_{r}}{\mu_{r,\;p}+2\mu_{r}}\right)\nabla H^{2},$$

where *r*_p_ (m) is particle radius and *µ_r, p_* is particle magnetic relative permeability.

### 2.2. Material properties

The couple system of Equations (1)–(8) is solved by COMSOL multiphysics software. Table 1 shows different properties of the four-layer artery model, including porosity, blood density, dynamic viscosity, and permeability. Usually nanoparticle solutions with about 10^6^–10^9^ particles are injected into the body for MDT. On the other hand, the particles are synthesized properly to prevent their interparticle interactions (Kempe & Kempe, 2010; Kong et al., 2012). In this study, it is assumed that 5000 ferroparticles are released in one-time step while their retention with the magnetic field is investigated. The particles are considered to be made of iron (III) oxide (Fe_3_O_4_) with a density of 5230 (kg/m^3^) and a relative magnetic permeability 4.1 (Lunnoo & Puangmali, 2015) within the diameter range of 100–2000 nm. These particles are released with uniform distribution at the artery inlet. However, their initial releasing position is subject to changes during the study. The studied drug/cargo is also considered to be made up of Fe_3_O_4_ within the diameter range of 10–50 µm with the same releasing conditions stated for particle cluster.

**Table 1.** Properties of different layers of the artery (Yang & Vafai, 2006).

	Thickness (µm)	Blood density (kg/m^3^)	Blood viscosity (Pa.s)	Porosity	Blood permeability (m^2^)
Lumen	6200	1057	3.7e-3	–	–
ET	2	1057	0.72e-3	5e-4	4.32e-21
Intima	10	1057	0.72e-3	0.983	2e-16
IEL	2	1057	0.72e-3	0.002	4.392e-19
Media	200	1057	0.72e-3	0.258	2e-18

## 3. Results and discussions

### 3.1. Validation

Yang & Vafai (2006) studied the four-layer model of arterial wall and obtained filtration velocity profiles for different boundary conditions along the lumen–ET interface. Due to the pressure difference between lumen and adventitia (the transmural pressure Δ*p* = lumen outlet pressure − adventitia pressure), an interstitial flow is generated inside the tissue (filtration velocity). However, this interstitial velocity is small due to the tissue porosity. Yang & Vafai (2006) obtained this velocity for three transmural pressures of 70, 120, and 150 mmHg, which are also studied herein.

The pressure gradients over the tissue and artery outlet lead to almost a constant blood flow velocity through the ET which is highly dependent on transmural pressure alteration. It is demonstrated that the simulation results are in a good agreement with Yang & Vafai’s work (2006). It should be mentioned that the number of grids for the simulations are increased to meet the mesh independency requirement (e.g. 214 400 grids for artery part). Supplemental Figure S1 shows the two-dimensional (2D) schematic model and the related boundary conditions, velocity profile in the artery and tissues, and filtration velocity.

In addition, Kim & Iglesias (1989) studied both experimentally and theoretically the deposition of particles inside a Y-shaped single branch tube with three different diameters of 3, 5, and 7 µm (representing a part of human airway) at three different flow rates of 4, 8, and 12 L/min. Kim’s work was also used to validate the present study. They set the dimensionless number of stokes as $\frac{\rho_{p}d_{p}^{2}\bar{u}}{36\mu R_{p}}$ where $\bar{u}$ (m/s) is average inlet air velocity and *R_p_* is inlet radius.

The laminar flow of air through a branch produced a stagnation point at both middle of the branch and near the turn points, and decreased the air velocity. Most of the particles deposited at the bifurcation point while the particles deposition was less at the turning points where the air flow rate was higher. Some particles adhered the main channel’s walls due to their near-wall initial positions. Simulation results for particle deposition also show a good agreement between numerical data and experimental values, validating the physics of fluid mechanics and particle tracing for our numerical models (Supplemental Figure S2; Kim & Iglesias, 1989).

Besides physical properties of the particles in fluid flow, the external magnetic field induces attraction forces to the particles at the stimulation zone (Equation (8)). Therefore, the magnetic field distribution generated within the media in our numerical model was verified against a theoretical analysis for the magnetic field distribution around a rectangular prism permanent magnet (Equation (9); Camacho & Sosa, 2013).

(9) $$B\;\left(y\right)=\frac{\mu_{0}M}{\pi }[\text{arctan}\frac{ab}{\left(y-c\right)\sqrt{a^{2}+b^{2}+{\left(y-c\right)}^{2}}}-\text{arctan}\frac{ab}{\left(y+c\right)\sqrt{a^{2}+b^{2}+{\left(y+c\right)}^{2}}}],$$

where *µ*_0_*M* is magnetization (0.87 ± 0.07 (T)), and ‘*a*’, ‘*b*’, and ‘*c*’ are magnet dimensions (herein *a* = 3 mm). The theoretical analysis of the magnetic field distribution around a magnet with 0.8 (T) field strength and is in a good agreement with our numerical results on MFS above the air surrounding magnet (Supplemental Figure S3). Following the verification of our numerical models for three physics of fluid flow, the particle tracing, magnetic field distribution, and MDT analysis through arteries are investigated in the following section.

### 3.2. Particles cluster and drug delivery

Following several numerical studies that used a magnet with a maximum field strength of ***B*** = 2 (T) for drug delivery analysis, we used 2 (T) permanent magnet to ensure that the maximal exposure of magnetic field to the artery does not exceed 0.4 (T) (Lunnoo & Puangmali, 2015). The ferroparticles released into the artery are trapped by the magnetic field at the targeted position. The key parameters considered in our simulation include MFS, magnet size, particle size, relative magnetic permeability of particles, and initial distribution of particles. Figure 1 shows the magnet geometry, the magnetic field, and the resultant particle trapping based on our numerical simulation.

**Figure 2.**
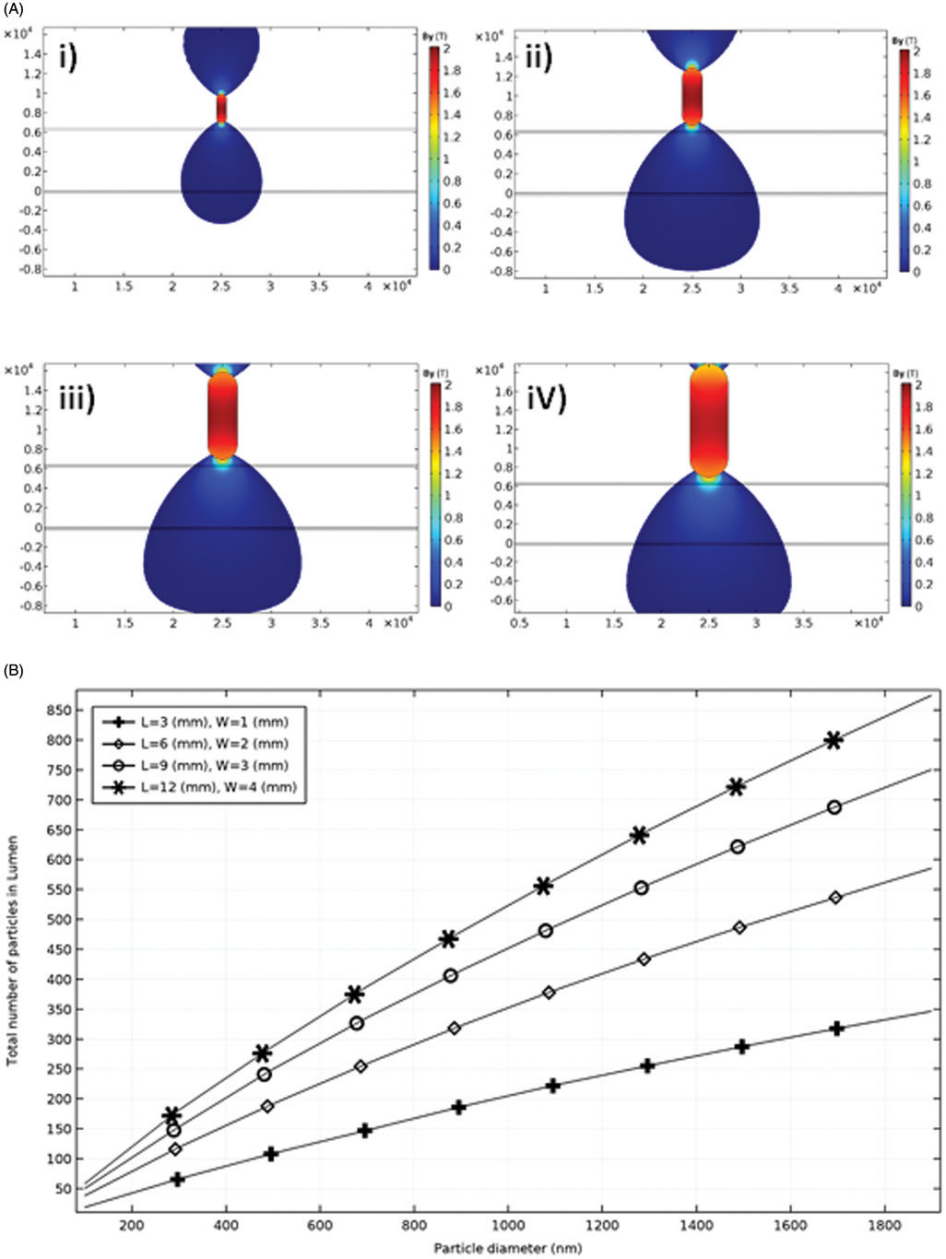
The effect of magnet size on the efficacy of particles trapping. (A) B_y_(T) around magnets of different size. (i) *L* = 3 mm, *W* = 1 mm; (ii) *L* = 6 mm, *W* = 2 mm; (iii) *L* = 9 mm, *W* = 3 mm; and (iv) *L* = 12 mm, *W* = 4 mm. (B) Total number of trapped particles for different magnet sizes.

First, the particle trajectory without any external magnetic field is studied where the fluid velocity near the lumen wall is low with a slow movement of particles through the lumen. Some particles remain near the ET layer because of the filtration and low boundary layer velocities. However, numerical results show that these effects are not persistent since most of these particles leave the lumen after 1 s while at least 10 s resident time is needed to make sure MDT stimulation is effective. Second, the magnetic field is applied at the target location (500 µm above the artery) to induce 0.4 (T) magnetic flux density inside the artery. When particles reach the desired place, they penetrate through the tissue and are trapped inside (Figure 1(B)). Among different parameters examined, the particle diameter and MFS are shown to be the most influential parameters on the deposition rate of particles. The results show that although magnetophoretic force and drag force do not have a linear relationship with particle diameter (Equations (6) and (8)), a linear relationship is observed for the effects of both MFS and particle diameter on particles retention (Figure 1(C)). For example, without changing the MFS, a one order of magnitude increase in particle size leads to an order of magnitude increase in the number of trapped particles. The effect of the magnet size on MDT performance and the resultant total number of trapped particles is shown in Figure 2 for four different sizes of the permanent magnet. Increasing the magnet size leads to covering a wider area of the tissue and in consequence leverages the potential of particles retention at the targeted place. Although the magnet size is effective in particles trapping, increasing the magnet size does not produce a linear increasing response in particles retention (Figure 2(B)). Thereby, a proper magnet size needs to be selected to achieve highest MDT performance.

**Figure 3.**
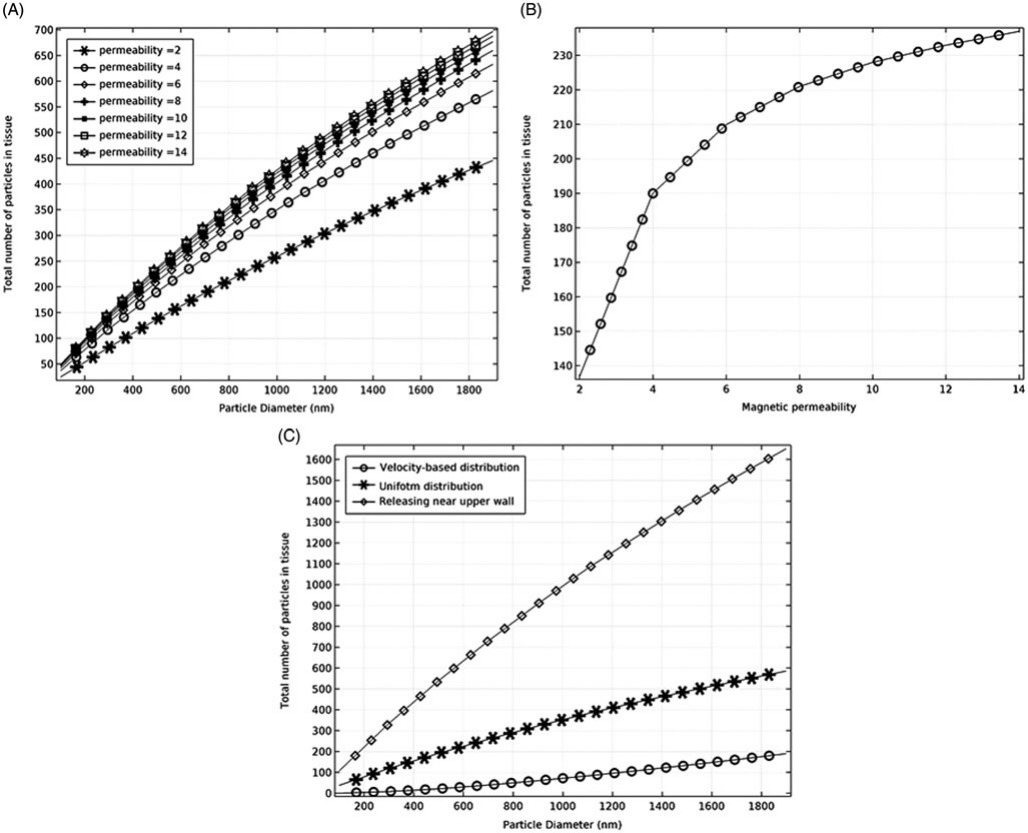
The effects of particles permeability and initial particle distribution on particles trapping. (A) The effect of magnetic permeability on particle retention. (B) The effect of magnetic permeability on particle trapping for 500 nm particles. (C) The effect of the initial distribution of particles on particle trapping.

Increasing the particles' magnetic permeability improves the particles retention at the tissue site (Figure 3(A)). The results show that particle permeabilities in the range of 4–12 do not have considerable effect on the particles trapping. For example, for particles with the diameter of 500 nm, increasing the particle permeability from 4 to 12 leads to an almost 0.8% increase in particles retention (Figure 3(B)). Furthermore, the effect of drug releasing distribution on the efficacy of particles retention was studied by simulating the particles released at three different initial positions, including the release at the inlet with a uniform distribution, release at a velocity-based distribution, and delivery near the upper wall (Figure 3(C)). The results show that the releasing mechanism of particles is very important in particles retention within the target tissue. Particles releasing near the upper wall results in a very successful particle trapping compared to the uniform or velocity-based releasing scenarios at the inlet.

### 3.3. Magnetic-based drug/cargo delivery

Utilizing micromotors for drug delivery in a single drug/cargo with various sizes (e.g. 50 µm Janus micromotors [Mou et al., 2014]) to a targeted site is considered as one MDT method with a tremendous potential for effective targeted drug delivery. External magnetic field can be applied to control the navigation of tubular, Janus, and stomatocyte cargoes to the targeted location (Gao et al., 2018; Mou et al., 2014; Patra et al., 2013; Xu et al., 2018). Herein, the movement of single Fe_3_O_4_ drugs/cargoes with different micrometer sizes and initial releasing locations in the arteries are studied to find out the feasibility of MDT method for artery drug/cargo delivery.

**Figure 4.**
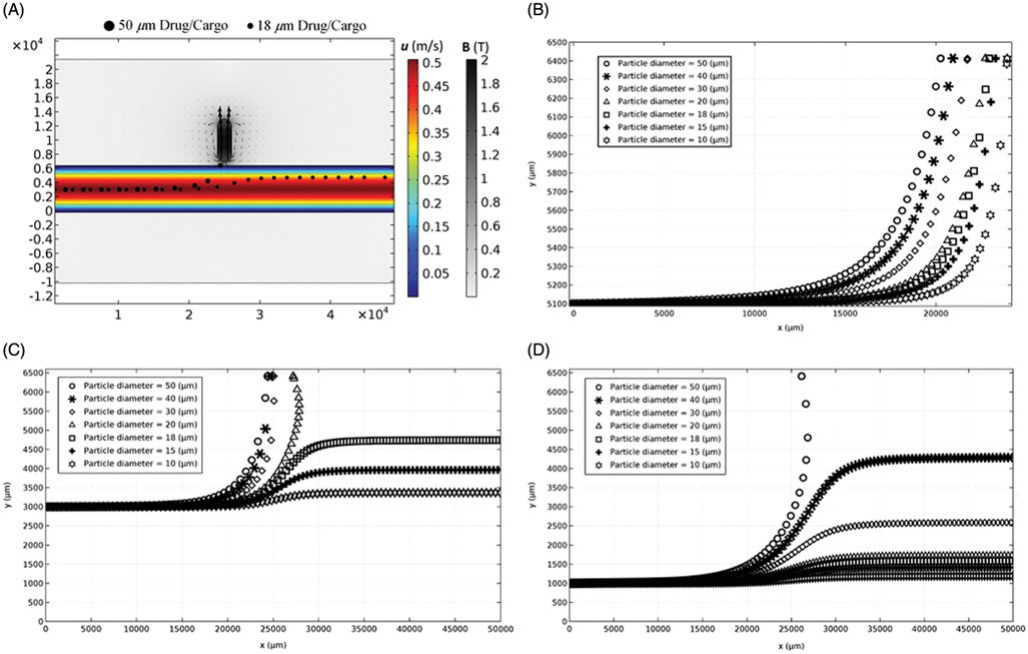
Particle position (*x*, *y*) moving through the artery with different diameters and initial positions at the inlet in the presence of external magnetic field. (A) The trajectories of single particles with 18 µm and 50 µm diameters through the artery (see Supplementary Movie 1). (B) Releasing a single particle with different diameters near the upper wall. (C) Releasing a single particle with different diameters at the middle of the artery. (D) Releasing a single particle with different diameters near the lower wall.

The driving force to transport particles through the artery is the blood drag force. The magnetophoretic force needs to overcome the drag force to be able to trap a particle at a desired site at the artery wall. Depending upon the magnitude of the magnetophoretic force, the magnetic force could either trap the particle on the wall or influence its trajectory near the magnet site (Figure 4(A) and Supplementary Movie 1). Moreover, since the drag force is lower near the artery wall than the middle of the artery, particles moving near the magnet wall are trapped within a wider range of particle diameters than other positions (Figure 4(B)–(D)). Also larger particles of 50 µm diameter showed higher retention chance near the magnet in different initial positions. The results show that the size of cargo, the magnetic field, and the initial releasing location need to be adjusted simultaneously to achieve a successful and noninvasive drug delivery through the arteries.

### 3.4. Luminal artery inflammation MDT

**Figure 5.**
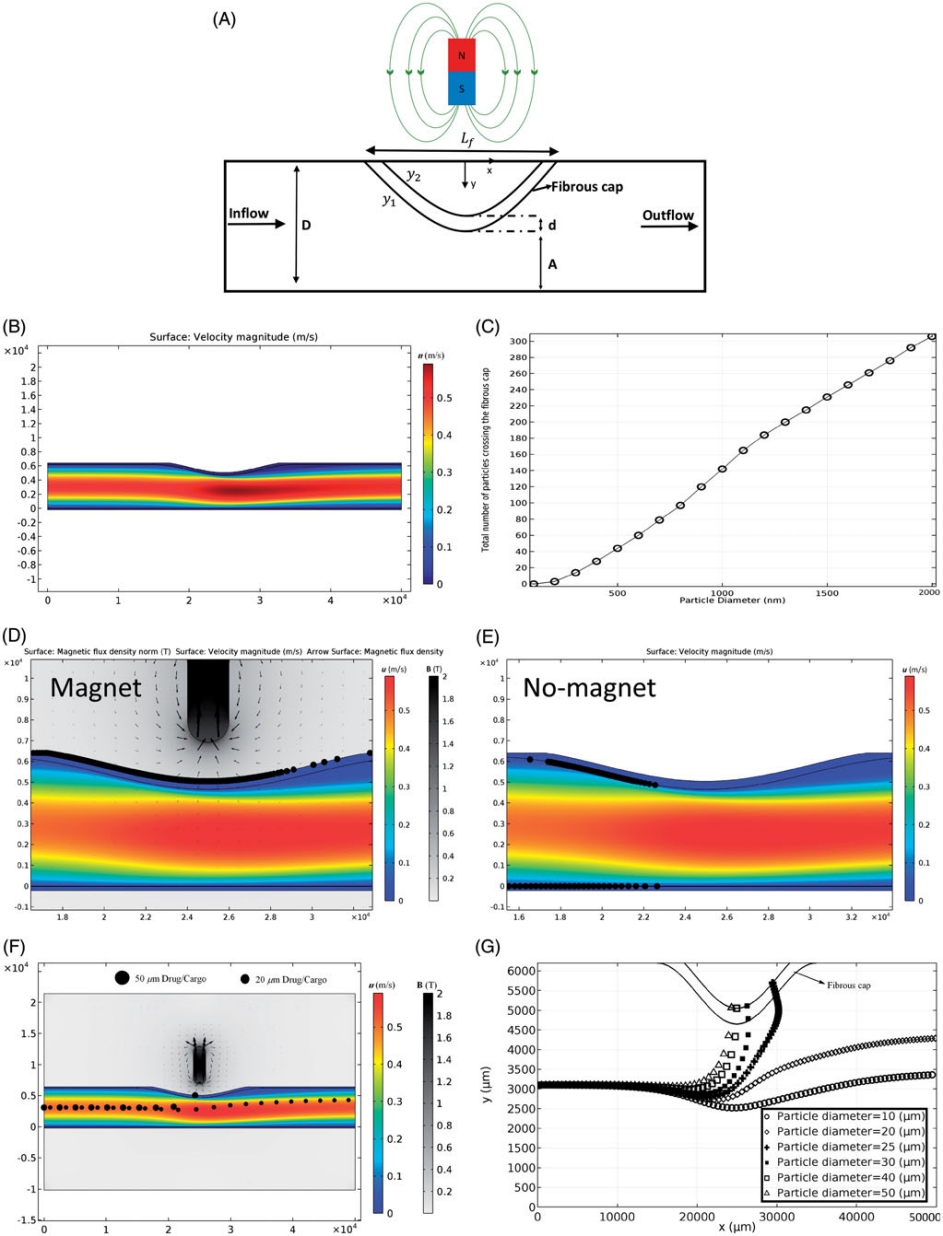
MDT through inflamed arteries. (A) The schematic illustration of the artery inflammation. (B) Blood flow through the inflamed artery. (C) The total number of drug particles passed the fibrous cap as a function of particle size. (D) The effect of magnetic field on a cluster of 2000-nm particles for artery inflammation MDT. (E) Particle retention in fibrous cap tissue in the absence of the magnetic field. (F) Comparing the behavior of 20 and 50 µm drugs/cargoes exposed to external magnetic field. (G) Trajectories of drugs/cargoes with different size in the presence of external magnetic field during their passage through the artery narrowing and interaction with the fibrous cap (Also see Supplementary Movie 2).

The application of MDT for the treatment of inflamed arteries using particle clusters and drug/cargo is also demonstrated (Figure 5). The four-layers artery model is used for the artery tissue; meanwhile, all boundary conditions for blood flow are similar to the ones shown in Table 1. The magnet has a length of *L* = 6 mm and a width of *W* = 2 mm, and a MFS of 2 (T). The inflammation shape (*y*_1_ and *y*_2_) is governed by a sinusoidal function as in Equations (10) and (11) (Li et al., 2006).

(10) $$y_{1}=\frac{D-A}{2}(1-\text{cos}\left(x\right)),$$

(11) $$y_{2}=\frac{D-A}{2}\left(1-\text{cos}\left(x\right)\right)-d,$$

where *D* = 6200 µm is the lumen diameter in the healthy part, *A* = 4650 µm is the lumen diameter in the maximal diseased part of the vessel, *L_f_* = 20000 µm is the length of the fibrous cap, and *d* = 400 µm is the fibrous cap thickness (Figure 5(A)).

Since most of the smooth muscle cells that create fibrous cap migrate from the media part of the tissue (Lee et al., 2017), the porosity of the fibrous cap is considered the same as the porosity of media. First, the cluster of 5000 ferroparticles with a diameter range of 100 nm to 2000 nm are released with a uniform initial distribution into the artery in the first time step and their behavior in the presence and absence of the external magnetic field is examined. The drag force increases at the inflamed region because of the local increase in blood velocity in this region (Figure 5(B)). On the other hand, the influential zone of the magnetic field is reduced at the inflamed region due to the increase in the distance between the magnet and blood flow region where the particles transport through the narrowing (Figure 5(D)). These two effects cumulatively reduce the efficacy of magnetic-based particle trapping at the fibrous cap. Our simulation results show that particles with the diameter of less than 100 nm have no penetration into the fibrous cap in the presence of the external magnetic field. Increasing the particle diameter above 100 nm enhances the chance of particles trapping (Figure 5(C)).

Some particles adhere undesirably to the fibrous cap or even the lower wall of healthy artery tissues at their narrowed part in the absence of external magnetic fields (Figure 5(E)). However, the drag force on adhered particles to the healthy tissue walls is not strong enough to be able to drive the particles into the tissue. In the presence of magnetic field, the magnetophoretic force pulls the particles locally toward the magnet next to the disease site and is strong enough to drive them into the tissue. Furthermore, the delivery capability of a single drug/cargo particle into the inflamed artery is investigated (Figure 5). The results show that the cargo diameter has a significant effect on the success of delivery to the diseased site (Figure 5(F)). Although the drag force is higher for larger cargo, the magnetophoretic force is dominant, and is able to trap the large cargo better than smaller ones (Figure 5(G) and Supplementary Movie 2). Compared to the small size of most cancer cargoes/drugs, larger sizes of cargoes are needed to recruit a successful MDT drug delivery strategy for the treatment of inflamed arteries.

## 4. Conclusions

This work presents a numerical model to investigate the efficacy of magnetic delivery of clusters of drug particles and drugs/cargoes in the luminal artery for the treatment of cancer and artery inflammation diseases. For the first time, the four-layer artery tissue model is employed and the crossing of magnetic particles through the tissue layers is considered. Moreover, the arterial fibrous cap that covers the inflamed region of the artery was subjected to MDT to assess the efficacy of this method for the treatment and healing of diseases such as atherosclerosis. This four-layer artery structure enabled observing the detailed interaction of particles and cargoes with tissue layers. The numerical results show that the architecture of the artery tissue and the fibrous cap affects the particles trapping on the wall and penetration into the tissue. Some released particles showed the tendency to adhere to the healthy porous ET. However, due to the low drag force, they have shown a less penetration to the tissue. In addition, for an inflamed artery and in the absence of external magnetic fields, some particles trap over fibrous cap and the lower wall of the artery due to the collision of particles and the increase in drag force as a result of the increase in blood velocity in this region.

The results of tracing the clusters of particles subjected to external magnetic field show that increasing the particles diameter from 50 to 2000 nm for MDT leads to a higher chance of trapping at the target place. Increasing the relative magnetic permeability of particles improved the chance of particles trapping at the target. However, the effect of particles permeabilities in the range of 4–12 on the efficacy of particles retention is trivial. Furthermore, the initial distributions of the released particles are shown to be important. The closer the particles are released with respect to the tissue wall, the better the particles are entraped. This is mainly because MFS is higher near the magnet which results in exerting a higher magnetophoretic force on particles in this region. Furthermore, the total number of trapped particles is shown to be linearly proportional to the MFS. Larger magnets cover a wider area of the artery tissue and result in better particle trapping at the desired site. However, an appropriate size of magnet needs to be selected since the magnet size has no linear relationship with the trapping efficiency.

The results of a single drug/cargo particle tracing show that the size and initial position of the cargo significantly affect its retention by the magnetic field. Increasing the drug/cargo diameter from 10 to 50 µm for magnetic-based drug/cargo delivery increases the retention chance by the magnetic field in the luminal artery. If the cargoes are released next to the artery wall in the presence of magnet, a wide range of drugs/cargoes size could be trapped by the magnetic field at the desired place. Our results show that a 50-µm drug/cargo (if permitted to be delivered and has the capability to penetrate the endothelial barriers) is highly capable of being trapped at the artery regardless of its initial position. We also use our numerical model to examine the magnetic-based cargo delivery to inflamed arteries. It is shown that a strong magnetophoretic force needs to be applied to deepen the influence zone of the magnetic field. Therefore, large magnetic particles (above 20 µm in diameter) need to be released into arteries for effective magnetic-based drug delivery and treatment of the artery inflammation using cargoes/drugs.

## Supplementary Material

Supplementary_Movie2.wmv

Supplementary_move1.wmv

SI-July_02_2018.docx
