# Supplementary material for: Delivery of magnetic micro/nanoparticles and magnetic-based drug/cargo into arterial flow for targeted therapy
Source: Drug Deliv. 2018 Dec 6;25(1):1963–73. doi: 10.1080/10717544.2018.1497106 (PMC6292362; doi:10.1080/10717544.2018.1497106)
Supplement: SI-July_02_2018.docx [file IDRD_A_1497106_SM3910.docx]

**Delivery of Magnetic Micro/Nanoparticles and Magnetic-Based Drug/Cargo into Arterial Flow for Targeted Therapy**

Mohammad K. D. Manshadi, Mahsa Saadat, Mehdi Mohammadi, Milad Shamsi, Morteza Dejam, Reza Kamali, Amir Sanati-Nezhad*

*** Corresponding Author:**

Amir Sanati-Nezhad, Email: [amir.sanatinezhad@ucalgary.ca](mailto:amir.sanatinezhad@ucalgary.ca)

**Supplementary Information.**

**Figure S1.** Schematic of the four-layer model of artery and **B)** the boundary conditions, **C)** Velocity profile inside the lumen and tissues, **D)** Comparing the filtration velocity results in this study and Yang’s data (Yang and Vafai, 2006).

**Figure S2**. Particle tracing in a Y-shaped single branch tube. Y-shaped tube**: A)** Model schematics**, B**, **C**, and **D)** Particle tracing for 7 µm particles moving through the Y-shaped single branch tube at t=0.005 s, t=0.05 s, and t=0.5 s, respectively, **C)** Comparison between experimental results of Kim’s work (Kim and Iglesias, 1989) and our numerical model.

**Figure A3.** Schematic of the studied magnet with almost 0.8 (T) field strength. **A)** Schematic illustration of the magnet, **B)** Numerical results of magnetic field. Its resultant magnetic field, **C)** Comparing the numerical and analytical results of the magnetic field.

**References**

Kim, C. S., & Iglesias, A. J. (1989). Deposition of inhaled particles in bifurcating airway models: I. Inspiratory deposition. *Journal of Aerosol Medicine, 2*(1), pp. 1-14.

Yang, N., & Vafai, K. (2006). Modeling of low-density lipoprotein (LDL) transport in the artery-effects of hypertension. *International Journal of Heat and Mass Transfer, 49*(5), pp. 850-867.
